# Preparation and characterization of chitosan/whey isolate protein active film containing TiO_2_ and white pepper essential oil

**DOI:** 10.3389/fnut.2022.1047988

**Published:** 2022-11-21

**Authors:** Ying Wang, Ji Wang, Jing Lai, Xin Zhang, Yongliang Wang, Yingchun Zhu

**Affiliations:** ^1^College of Food Science and Engineering, Shanxi Agricultural University, Jinzhong, China; ^2^Shanxi Food Industry Institute, Taiyuan, China

**Keywords:** active biological film, structure characterization, barrier performance, antioxidative properties, antibacterial properties

## Abstract

Active packaging films are designed to improve quality and extend the food shelf life by incorporating functional active ingredients into biopolymer films. This study developed a bioactive film based on chitosan (CS) and whey isolated protein (WPI) incorporated with 0.01 wt% TiO_2_ and 0.1 wt% white pepper essential oil (WPEO). The physicochemical properties of the prepared film were also evaluated comprehensively. The results showed that water solubility and water vapor permeability of the film incorporated with TiO_2_ and WPEO were 25.09% and 0.0933 g mm m^–2^ h^–1^ KPa^–1^, respectively, which were significantly higher than those of other films (*P* < 0.05). In addition, the UV barrier properties of films incorporating TiO_2_ and WPEO have improved. The films were characterized by Fourier transform infrared (FTIR) and scanning electron microscopy (SEM). The FTIR results showed interactions between TiO_2_ and WPEO with CS/WPI compound, and the SEM results indicated a good incorporation of TiO_2_ into the composite films. The antioxidative and antibacterial properties of films were significantly enhanced by incorporating WPEO. According to results, the developed biocomposite film can be considered as a packaging material.

## Introduction

In recent years, the development of biodegradable films based on natural and renewable biopolymers for food packaging has attracted increasing attention (1, 2). Some environmentally friendly and biodegradable biopolymers such as polysaccharides, proteins, and lipids have been used to produce food packaging films. These packaging films not only provide moisture and gas barriers for food products, but also serve as carriers of bioactive substances (nanoparticles and essential oils) with antibacterial and antioxidant activity (3, 4). Chitosan/sodium alginate and chitosan/corn starch films were also proven to have good water vapor barrier and extensibility (5, 6). Previous studies have shown good water resistance of kefir/whey isolated protein (WPI) films and apple pectin/tapioca starch films incorporated with lauric oil and oleic acid (7, 8). However, Wang et al. (6) reported that sodium alginate/chitosan film have good water vapor barrier and UV barrier, but its tensile strength (TS) and water resistance are poor. In addition, antioxidant and antibacterial properties were significantly enhanced in biopolymer films incorporating nanoparticles and essential oils (EOs), such as peppermint EO (2), ginger EO (9), TiO_2_ nanoparticles (1, 7), and zinc oxide nanoparticles (8, 10).

Chitosan (CS) and WPI are considered as promising film-forming material owing to their excellent film-forming ability and biodegradability (10–12). However, single material films such as chitosan films and WPI films have poor water vapor barrier and mechanical properties (7, 8, 11). CS and WPI have been widely used to develop composite films, such as CS/WPI films incorporating modified TiO_2_ nanoparticles with sodium laurate (13), and chitosan/whey protein-based film containing nano-TiO_2_ and *Zataria multiflora* EO (14). Some studies have demonstrated that combining two or more biopolymers has considerably improved the mechanical properties, water resistance, and UV-barrier properties of composite films (6, 14, 15). Tavares et al. (11) showed that TS of CS/WPI film increased by 212.1% compared to CS film and water vapor permeability (WVP) decreased by 15.9% compared to CS film. In addition, the incorporation of desirable compatible nanomaterials or bioactive substances into biopolymer matrix not only improves physical properties of the films, but also enhances the antioxidant and antibacterial properties of the films, improving food quality and extending shelf life (11, 13, 16).

Due to its non-toxicity, inexpensiveness, and stability, TiO_2_, as a type of nanoparticles, is usually used for food packaging to enhance the functional properties of films such as radiation resistance and antimicrobial activity (17, 18). Recent research has reported that the incorporation of TiO_2_ nanoparticles into polymeric films can considerably improve the mechanical properties and stability such as TS, water resistance, and thermal property (13, 19). Furthermore, nano-TiO_2_ has been demonstrated to provide protection against food-borne microorganisms in the presence of UV radiation (20). Zhang et al. (16) reported that the incorporation of TiO_2_ enhanced the mechanical properties and gas permeation barrier of CS films and the CS/nano-TiO_2_ composite film effectively inhibited bacteria, fungi, and molds. Enescu et al. (21) evaluated the migration of TiO_2_ in CS films and found that TiO_2_ migrated into various food simulants mainly in the form of titanium ions, and the amount of TiO_2_ migrated into the food simulants was negligible. Similarly, Alizadeh-Sani et al. (22) investigated the migration characteristics of TiO_2_ from WPI films during mutton packaging and found that TiO_2_ was detected in mutton samples stored for 15 days at less than 0.064 ppb, which was well below the Food and Drug Administration (FDA) recommended limit. The FDA has approved the use of TiO_2_ in foods as a color additive (E171) at levels up to 1%. In addition, it was found that most nanoparticles in composites formed agglomerates larger than 100 nm in diameter, so that nanoparticles incorporated into polymers tend to aggregate and remain firmly embedded in the polymer matrix and are less likely to migrate (23).

Essential oils are a rich source of various bioactive compounds such as phenols, terpenes, and terpenoids, and their unique antimicrobial and antioxidant properties have promoted their wide applications in the food industry (24, 25). Studies by Almasi et al. (26) and Amalraj et al. (27) reported that biopolymer films containing EOs significantly inhibited the growth of *Staphylococcus aureus* and *Escherichia coli*. Some studies by Asadi (28) and Elfahdi (29) demonstrated that the dominant bioactive components of pepper EO were caryophyllene and limonene. Wang et al. (30) detected caryophyllene, limonene and 3-carene in pepper EO. In addition, Li et al. (31) analyzed different varieties of pepper EOs from five regions in southern China and found that they exhibited different antioxidant and antibacterial properties. The strong antioxidant and antibacterial activity of pepper EO is due to its high concentration of monoterpenes (32, 33). Thus, our major interest is to explore the potential of the incorporation of WPEO and nano-TiO_2_ into CS/WPI films as a multifunctional packaging material with the satisfactory antioxidant, antimicrobial, mechanical properties, and UV barrier properties.

In this study, active film by incorporating WPEO and nano-TiO_2_ into CS/WPI polymer matrix was developed. The effects of TiO_2_ and/or WPEO on the physicochemical and structural characteristics of CS/WPI films were compared. And the physical, structural, antioxidant, and antimicrobial properties of the films were evaluated.

## Materials and methods

### Materials

Chitosan (deacetylation degree of 95%) and nano-TiO_2_ (particle size of 5–10 nm, purity of ≥99.8%) was purchased from Shanghai Acmec Biochemical Co., Ltd. (Shanghai, China). WPI (protein content of >90 wt.%) was obtained from Beijing Jialikangyuan International Trade Co., Ltd. (Beijing, China). White pepper essential oil (WPEO) was provided by Tianjin Chunhe Technology Development Co., Ltd. (Tianjin, China). The 2,2-diphenyl-1-picrylhydrazyl (DPPH) and 2,2′-Azinobis-(3-ethylbenzthiazoline-6-sulphonate) (ABTS) were obtained from Beijing Solarbio Science & Technology Co., Ltd. (Beijing, China). Ethanol, glycerol, and glacial acetic acid were obtained from Tianjin Chemical Reagent Factory (Tianjin, China). *E. coli* ATCC 8739NA, *Listeria monocytogenes* ATCC 19114, *S. aureus* ATCC 6538, and *Salmonella* CMCC 50041-16 were provided by Bioengineering Laboratory, College of Food Science and Engineering, Shanxi Agricultural University (Shanxi, China). The chemicals used for the experiments were of analytical grade.

### Preparation of films

Chitosan solution was prepared by adding chitosan (1% w/v) to glacial acetic acid solution (1% v/v) with constant stirring, then heated at 60°C for 40 min. The 0.4 g of WPI was completely dissolved in 100 ml distilled water and the pH was adjusted to 8 with 1 mol/L NaOH. The WPI solution was heated at 85°C for 30 min with constant stirring to denature the WPI. The CS solution and WPI solution were fully mixed, and glycerol (1% w/v in mixed solution) was added to the mixed solution and the mixture was stirred for 1 h. TiO_2_ nanoparticles (0.01% w/v in the mixed solution) were slowly added into the CS/WPI solution and stirred with the homogenizer (FA25, Shanghai Fluke Fluid Machinery Manufacturing Co., Ltd.) at 1,000 r/min for 10 min and sonicated for 20 min using an Ultrasonic Cleaner (JY92-N, Ningbo Biotechnology Co., Ltd.) until the TiO_2_ was well dispersed. Finally, WPEO (0.1% w/v in the mixed solution) was added to the mixed solution, and the solution was completely homogenized for 10 min using a homogenizer. The film-forming solution was poured and spread in an acrylic square plate (12 cm × 12 cm) and dried by an oven at 25 ± 2°C for 24 h (34).

### Detection of film physical properties

#### Thickness

The film thickness was measured at five random locations on each sample by using a digital micrometer (Shanghai Minet Industrial Co., Ltd.) with an accuracy of 0.001 mm.

#### Water solubility

Water solubility (WS) was determined by previously reported method (20). The film samples were dried in an oven at 105°C to a constant weight (W_1_), and immersed in 100 ml distilled water at room temperature for 24 h, and re-dried at 105°C to a constant weight (W_2_). The WS of films was calculated using Eq. 1.


(1) $$WS\left(\%\right)=\frac{W_{1}-W_{2}}{W_{1}}\times 100.$$

#### Water vapor permeability

The 2 g of anhydrous calcium chloride was placed into a small beaker (50 ml) and covered with a piece of film, and then the beaker containing anhydrous calcium chloride was placed into a desiccator with 75% relative humidity (RH) (maintained by NaCl saturated solution). The total weight of the beaker and anhydrous calcium chloride was determined after 24 h. The WVP of films was calculated using Eq. 2.


(2) $$W\,V\,P\,\left(g•m\,m•m^{-2}•h^{-1}•K\,P\,a^{-1}\right)=\frac{\Delta \,m\times d}{t\times A\times \Delta \,P}$$

where *Δm* represented the increased weight of the beaker (g); *d* represented film thickness (mm); *t* was 24 h; *A* represented permeation area of film (m^2^), and *ΔP* was 3.168 KPa at room temperature.

#### Mechanical properties

Tensile strength (MPa) and elongation rate at break (Eb, %) were measured according to previously reported method (10) with minor modification. Each film was cut into rectangles (100 mm × 15 mm) and then fixed between the grips of TA. XT Plus texture analyzer (Stable Micro Systems Ltd., UK) with a crosshead speed set as 20 mm/min. At least five replicates were performed for each sample.

#### Color and opacity

The L* (lightness), a* (redness), and b* (yellowness) values of films were determined using a colorimeter (CM-5, Konica Minolta, Japan). The instrument was standardized using a light trap and white tile. Using illuminant D 65 with a 10° observer and a 10 mm diameter aperture. The L*, a*, and b* values of five records for each film were averaged, and the color difference (ΔE) and whiteness index (WI) were calculated using Eqs 3, 4.


(3) $$\Delta E=\sqrt{\left({\left(\Delta L^{*}\right)}^{2}+{\left(\Delta a^{*}\right)}^{2}+{\left(\Delta b^{*}\right)}^{2}\right)}$$

(4) $$WI=100-\sqrt{{\left(100-L^{*}\right)}^{2}+{\left(a^{*}\right)}^{2}+{\left(b^{*}\right)}^{2}}$$

Film samples were cut into rectangles (3 cm × 1 cm), and placed in cuvettes. The UV-Vis spectra within 200–800 nm were recorded with a UV-vis spectrophotometer (Cary 4000, Agilent Co., Ltd., USA), and a blank cuvette was used as reference. The opacity value of films was calculated using Eq. 5.


(5) $$\text{Opacity}=\frac{A_{600}}{x}$$

where *A*_600_ was the film absorbance at 600 nm, and *x* was the film thickness (mm).

### Characterization of films

#### Fourier transform infrared analysis

The Fourier transform infrared (FTIR) spectra of each film were recorded using a Tensor 27 FTIR spectrometer (Bruker, Germany). Measurements were performed at room temperature within the wavelength range of 4,000–400 cm^–1^ with 16 scans at a resolution of 4 cm^–1^.

#### Scanning electron microscopy

The microstructures of surface and cross-sections of the films were observed by scanning electron microscopy (SEM) (JSM-7500F, Japan) at 12.0 kV accelerating voltage. The films were fractured in liquid nitrogen, fixed on a metal stake and sputtered with gold. Then the images were recorded at 100× and 1,000× magnifications.

#### Contact angle measurements

The water contact angle (WCA) of the films was measured using a contact angle meter (OCA20, Dataphysics, Germany). A drop of ultrapure water (about 5 μl) was placed on the surface of each film (40 mm × 40 mm). After 30 s of exposure, the drop images were captured by a CCD camera and conveyed to the computer for the measurement. Five different random positions of each film were tested, and the mean values were calculated.

#### Antioxidant property

The antioxidant property of films was evaluated through DPPH and ABTS radical scavenging assays. DPPH radical scavenging activity was determined by the previously reported method (9) with minor modification. Specifically, 2 ml of film solution was mixed with 2 ml of DPPH solution, and the absorbance at 517 nm was measured after 30-min reaction in the dark at room temperature. DPPH radical scavenging activity was calculated using Eq. 6.


(6) $$\mathrm{DPPH}\mathrm{radical}\mathrm{scavenging}\mathrm{activity}\left(\%\right)=\left(1-\frac{A_{i}-A_{j}}{A_{0}}\right)\times 100$$

where *A*_0_ is the absorbance of the mixture of distilled water and DPPH solution; *A*_*i*_ is the absorbance of the mixture of film solution and DPPH solution; and *A*_*j*_ is the absorbance of the mixture solution of anhydrous alcohol and film solution.

The ABTS radical scavenging activity was determined by the method reported by Hashemi and Mousavi Khaneghah (35). ABTS (7 mM) and potassium persulfate (2.45 mM) stock solutions were used. The absorbance at 734 nm was measured using a spectrophotometer (UV-1100, Mepada Shanghai Instruments Co., Ltd., China).

#### Antibacterial property

The agar disc diffusion method was employed for determining the antimicrobial activity of films (17). *E. coli*, *L. monocytogenes*, *S. aureus*, and *Salmonella* were incubated in nutrient broth at 37°C overnight. The bacterial solution was diluted with saline (0.85% NaCl) to 10^6^ CFU/ml of bacterial concentration. Then, 100 μl of bacterial suspension was spread evenly on the agar plate, and the prepared films were aseptically cut into 10 mm diameter discs and placed on the agar plates. Finally, the plates were incubated at 37°C for 24 h. Afterward, the diameter of the bacterial inhibition zone was measured by using calipers.

#### Release rate of white pepper essential oil

The EO release rate was determined according to the previously reported method (36). Three standard food simulants were used with distilled water as water-based food simulant, 50% (v/v) ethanol as oil-in-water emulsion and alcohol food simulant, and 95% (v/v) ethanol as fatty food simulant. One gram film sample was immersed in each of three centrifuge tube respectively containing 30 ml one type of food simulant with constant stirring. One milliliter of solution was pipetted from each of the three tubes at regular intervals. The centrifuge tubes were supplemented with each food simulant to original amount (30 ml). The absorbance at 276 nm was measured using a UV-Vis spectrophotometer. The maximum absorption peak of WPEO was measured by full wavelength scan before detection, the standard curve of absorbance and EO concentration was plotted.

### Statistical analysis

The statistical analysis of data was performed using Statistic 8.1 software (Statsoft Inc., USA). Origin 8.0 software (Origin Lab Corporation, USA) was used for plotting. The data were expressed as the mean value ± SD (standard deviation) of the triplicates. *P* < 0.05 was considered as statistically significant. The experiment was conducted in three biological and technical parallels.

## Results

### Physical properties of films

#### Thickness

Film thickness greatly affects the light transmittance and mechanical strength of films. As shown in Table 1, the thickness of CS film was 0.016 mm. The addition of WPI, TiO_2_, and WPEO into the CS matrix significantly increased the thickness of films (*P* < 0.05). Similar results were observed in CS/WPI film (12) and CS film incorporated with ginger EO (9). This result might be attributed to the increase in solid content in the film-forming solution, which remained after the film dehydration (13). The thickness difference between CS/WPI film and CS/WPI/TiO_2_ film was not significant (*P* > 0.05). The thickness of CS/WPI/WPEO film and CS/WPI/WPEO+TiO_2_ film was significantly higher (*P* < 0.05) than CS film, CS/WPI film and CS/WPI/TiO_2_ film. The possible reason for the greater thickness was that the former had more porous microstructure.

**TABLE 1:** The parameters of thickness, WS, WVP, TS, and Eb in prepared films.

Films	Thickness (mm)	WS (%)	WVP (g mm m^–2^h^–1^ KPa^–1^)	Tensile strength (MPa)	Elongation at break (%)
CS	0.016 ± 0.002^d^	27.61 ± 0.13^a^	0.1035 ± 0.0005^a^	17.41 ± 0.24^d^	75.64 ± 0.77^d^
CS/WPI	0.019 ± 0.003^c^	26.64 ± 0.13^b^	0.1013 ± 0.0003^a^	20.74 ± 0.18^c^	82.27 ± 0.90^c^
CS/WPI/TiO_2_	0.020 ± 0.002^c^	25.55 ± 0.16^c^	0.0935 ± 0.0003^b^	22.32 ± 0.12^a^	88.67 ± 1.02^b^
CS/WPI/WPEO	0.021 ± 0.001^b^	25.18 ± 0.16^c^	0.0945 ± 0.0005^b^	19.02 ± 0.16^b^	94.48 ± 1.06^a^
CS/WPI/WPEO/TiO_2_	0.024 ± 0.003^a^	25.09 ± 0.19^c^	0.0933 ± 0.0002^b^	21.23 ± 0.19^ab^	92.69 ± 0.66^a^

Data are expressed as mean ± SD. Different letters (a–d) in each column indicate significant differences among films (*P* < 0.05).

#### Water solubility

As shown in Table 1, the WS of CS film was 27.61%, which was higher than the value 20.98% reported by Chang et al. (17) and 23.43% reported by Li et al. (37). While Ren et al. (5) reported the solubility 32.73% for CS film, and this may be due to differences in molecular weights, degree of deacetylation and content of CS. CS film has higher WS than other films, which might be due to the abundant hydrophilic groups, especially -OH and -NH_2_ in CS molecules (12). The WS value of the CS/WPI film was significantly decreased to 26.64% (*P* < 0.05), which might be due to the coalescence between CS and WPI, thus reducing the interaction between film and water molecules. Similarly, Tavares et al. (11) also reported the solubility of CS/WPI film (14.58%) was lower than that of CS film (41.82%).

Moreover, WS values of CS/WPI/TiO_2_, CS/WPI/WPEO, and CS/WPI/WPEO+TiO_2_ films were further significantly decreased to 25.09–25.55% (*P* < 0.05). The reduction in WS of composite films might be related to the compact structures and strong bonds generated from the interactions among TiO_2_, WPEO, and CS/WPI polymer matrix. de Menezes et al. (38) reports that the solubility of chitosan/cassava starch with 1% TiO_2_ was significantly smaller than CS film by 23%. This indicates that TiO_2_ can combine with the composite substrate to form compact network structure, thus preventing water molecules from entering into the films (7, 17). In addition, the intermolecular interactions between WPEO and CS/WPI may limit the formation of hydrophilic bonds between the hydroxyl groups and water molecules, thus leading to a decrease in the affinity to water of the composite film (27, 34).

#### Water vapor permeability

Water vapor permeability values of prepared films were presented in Table 1. The WVP value of CS film was 0.1035 g mm m^–2^ h^–1^ KPa^–1^, while that of CS/WPI film was 0.1013 g mm m^–2^ h^–1^ KPa^–1^. The lower WVP value of CS/WPI film might be attributed to the formation of hydrogen bonds between the CS and WPI, in turn resulting in the reduction of hydrophilic groups in WPI (11).

Compared with that of CS film and CS/WPI film, the WVP value of CS/WPI/TiO_2_, CS/WPI/WPEO and CS/WPI/WPEO+TiO_2_ films was significantly decreased to 0.0933–0.0945 g mm m^–2^ h^–1^ KPa^–1^(*P* < 0.05). This result may be associated with the fact that the formation of hydrogen bonds between the CS/WPI polymer matrix and TiO_2_ or WPEO caused the reduction in hydrophilic groups in CS and WPI, which could be further explained by the possibility that TiO_2_ nanoparticles may interact with hydrophilic -OH and -NH groups in CS, thus lowering the hydrophilic groups responsible for the sorption of water vapor on composite films surface (13, 18). Our results were consistent with the reported by Zhang et al. (19), who demonstrated that WVP of the CS film decreased from 1.8472 g mm m^–2^ h^–1^ KPa^–1^ to 1.6167 g mm m^–2^ h^–1^ KPa^–1^ with the incorporation of TiO_2_. Moreover, the WVP of the films incorporating WPEO was also significantly lower than that of CS film and CS/WPI film, which might be due to the hydrogen bonding and covalent interactions between composite substrate and the compounds in EO. These interactions may block the hydrophilic groups from forming hydrophilic bonds, thereby reducing the entry of water molecules into the composite films (39). Li et al. (40) also reported a significant reduction in WVP of 34.4% by incorporating 0.5% orange peel EOs into chitosan/fish skin gelatin film.

#### Mechanical properties

Good mechanical properties of packaging films are crucial for food packaging and storage. The mechanical properties of film are evaluated by TS and Eb. The mechanical properties of prepared films were shown in Table 1. The TS value of CS film was 17.41 MPa, and its Eb value was 75.64%, which were higher than 14.21 MPa and 44.7% reported by Lan et al. (1). The TS and Eb values of CS/WPI composite film reached 20.74 MPa and 82.27%, respectively, which were significantly higher than those of CS film (*P* < 0.05). TS value of CS/WPI film increase might result from the covalent cross-linking between intermolecular disulfide bonds induced by the thermal denaturation of WPI (11). In addition, the gel formation during the heating process resulted in an increase in the viscosity of the film-forming solution, and thus the CS/WPI film became softer and exhibited a significant improvement in the Eb value.

The incorporation of TiO_2_ nanoparticles caused a significantly increased TS value 22.32 MPa and Eb value 88.67% of CS/WPI/TiO_2_ film. The increase in TS value might be attributed to the high surface energy of TiO_2_ and the strong interfacial interaction between TiO_2_ and CS/WPI (41), leading to an increase in the density and rigidity of the three-dimensional network structure, eventually increasing mechanical strength. Our results were in line with Zhang et al. (13) who reported an increase in TS from 16.18 to 18.04 MPa and an increase in Eb from 158.55 to 177.57% for CS/WPI film incorporating TiO_2_.

The Eb value of the films added with WPEO was higher than that of the films without additional WPEO, and Eb value of CS/WPI/WPEO film and CS/WPI/WPEO+TiO_2_ film was 94.48 and 92.69%, respectively. Similarly, Amalraj et al. (27) reported that Eb of polyvinyl alcohol/gum arabic/chitosan film incorporated with black pepper EO and ginger EO were significantly increased (*P* < 0.05), and reaching 97.40 and 94.62%, respectively. This indicated that EOs incorporated into the CS/WPI solution act as a film plasticizer. This can be explained by the fact that EO penetrates into the biopolymer matrix to decrease the strength of intermolecular interaction, but increase the plasticity and extensibility of the film (27, 42). Our dates showed that TS of CS/WPI/WPEO film was decreased by 8.29% compared to CS/WPI film, which might be mainly attributed to the partial replacement of the strong intermolecular interaction in the CS/WPI matrix with the weak interaction between EOs and polymer in the network structure, thus reducing the cohesion of polymer network (41). Our results of TS values were in line with the reports by Xu et al. (42) and Alizadeh-Sani et al. (34), who observed that the incorporating of EOs significantly decreased TS of composite films.

### Color and transparency of films

To understand the optical properties of the films, we investigated color parameters L* (lightness), a* (redness), b* (yellowness), ΔE (total color difference), WI (whiteness index), and opacity (Op). As shown in Table 2, the a* values were negative and the b* values were positive for the two films added with WPEO, indicating that the addition of WPEO resulted in the turning of the film color to yellow-green. Of all the films, CS/WPI/TiO_2_ film exhibited the highest L* value (39.29) and WI value (39.22). The significantly increased whiteness and brightness of the composite film might be attributed to the addition of white nano-TiO_2_. Our results were in accordance with Lan et al. (1) who reported an increase in L* value range from 78.94 to 85.66 for CS film incorporated with TiO_2_. The ΔE values of the films added with WPI, TiO_2_, and WPEO were significantly lower (*P* < 0.05), indicating that these additives increased the color uniformity of the films.

**TABLE 2:** The parameters of color and opacity in films.

Films	L*	a*	b*	ΔE	WI	Opacity (mm^–1^)
CS	35.12 ± 0.49^c^	−0.42 ± 0.06^a^	−0.22 ± 0.02^b^	58.24 ± 0.47^a^	35.12 ± 0.34^c^	3.62 ± 0.02^d^
CS/WPI	36.67 ± 0.17^b^	−0.72 ± 0.01^b^	−0.69 ± 0.07^c^	56.62 ± 0.15^b^	36.67 ± 0.16^b^	8.61 ± 0.03^c^
CS/WPI/TiO_2_	39.29 ± 0.54^a^	−1.46 ± 0.03^c^	−0.86 ± 0.05^c^	54.13 ± 0.42^c^	39.22 ± 0.26^a^	17.4 ± 0.03^ab^
CS/WPI/WPEO	37.27 ± 0.43^b^	−2.55 ± 0.02^d^	1.25 ± 0.04^a^	55.73 ± 0.33^b^	37.25 ± 0.23^b^	16.7 ± 0.04^b^
CS/WPI/WPEO/TiO_2_	38.76 ± 0.55^a^	−1.92 ± 0.05^e^	1.85 ± 0.04^a^	54.12 ± 0.20^c^	38.70 ± 0.33^a^	17.9 ± 0.04^a^

Data are expressed as mean ± SD. Different letters (a–d) in each column indicate significant differences among films (*P* < 0.05).

The pure CS film was transparent, whereas CS/WPI film was translucent. The opacity of CS/WPI/TiO_2_ film was 17.4, which was significantly higher than that of CS film (3.62) and CS/WPI film (8.61), probably because of the light-scattering effect of the TiO_2_ nanoparticles (9). The white color of the CS/WPI/TiO_2_ film is related to the inherent properties of TiO_2_. Consistent with our study, previous research has also reported that nano-TiO_2_ addition reduced the transparency of the WPI-Kefiran composite films (7). In this study, the opacity of CS/WPI/WPEO film and CS/WPI/WPEO/TiO_2_ film was increased significantly (*P* < 0.05), which might be explained by the fact that the addition of EOs resulted in more pores in composite films, thus scattering light (26). The similar observation has been reported that CS film opacity was increased by 11.98% incorporating rosemary EO (43).

**FIGURE 1:**
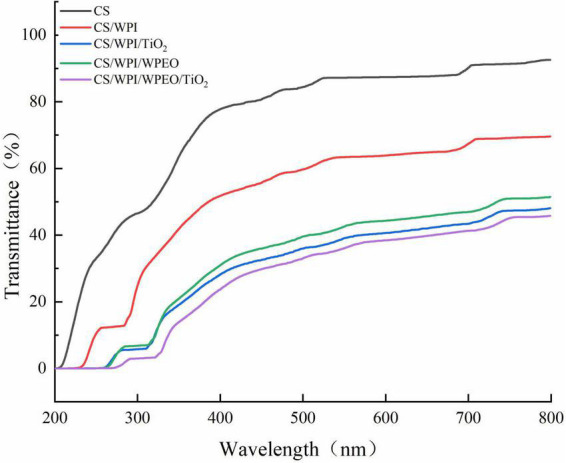
UV–vis light transmittance of films.

Foods are prone to spoilage when they are exposed to UV-vis light (12). Therefore, UV-vis light barrier property is crucial for food packaging films. As shown in Figure 1, CS film exhibited the highest UV-vis light transmittance. In comparison to CS film, the films added with TiO_2_ and WPEO had remarkably lower UV-vis light transmittance. Similarly, Zhang et al. (19) also reported a significant decrease in UV-vis transmittance for CS film incorporated with TiO_2_ and black plum bark extract. This phenomenon might be due to high UV absorption ability of polyphenolic compounds (41). The light scattering effect of nano-TiO_2_ may lead to the further interactions between visible light and CS/WPI/TiO_2_ composite film to reduce the light transmittance of the film, thus effectively protecting the film from UV-vis light (16).

It should be noted that UV light transmittance of CS/WPI/WPEO, CS/WPI/TiO_2_, and CS/WPI/WPEO+TiO_2_ composite films was nearly zero within the range of 200–300 nm (Figure 1), indicating the incorporation of functional substances such as EOs and nano-TiO_2_ into pure or mixed biopolymer films could significantly improve the UV-vis light barrier properties, and thus these substances can be used as potential UV shielding materials (32, 44). Several previous studies have also reported that CS films incorporating active substances (e.g., plant extracts, TiO_2_, etc.) have good UV-vis light barrier properties (12, 19).

### Characterization of films

#### Fourier transform infrared

**FIGURE 2:**
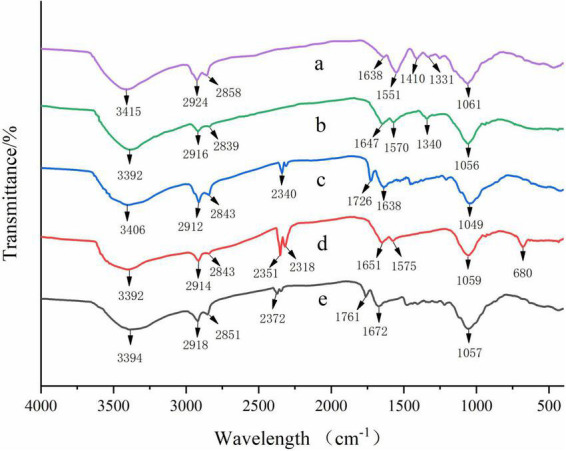
Fourier transform infrared spectra of CS (a), CS/WPI (b), CS/WPI/WPEO (c), CS/WPI/TiO_2_ (d), and CS/WPI/WPEO/TiO_2_ (e) films.

Fourier transform infrared spectra of the prepared films were presented in Figure 2. A broad band was observed near 3,400 cm^–1^, which was attributed to the stretching vibration of N-H and O-H groups in all films (5). The band observed near 2,920 and 2,850 cm^–1^ corresponded to C-H stretching vibration (45). The absorption peaks of C = O stretching were detected near 1,650 cm^–1^, and the peak located at around 1,050 cm^–1^ was related to the C-O-C stretching vibration (17).

**FIGURE 3:**
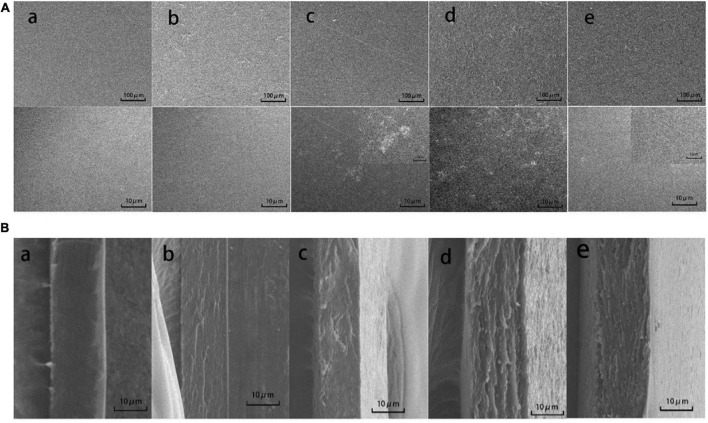
Scanning electron microscopy photographs of the surfaces **(A)** and cross-sections **(B)** of CS (a), CS/WPI (b), CS/WPI/WPEO (c), CS/WPI/TiO_2_ (d), and CS/WPI/WPEO/TiO_2_ (e) films.

In the CS film, the absorption peak at 1,551 cm^–1^ was related to the N-H bending vibration. The bands observed at 1,410 and 1,331 cm^–1^ were due to the scissoring vibrations of NH_2_ groups and the C-N stretching vibration (6, 46).

The peaks at 3,415, 1,638, and 1,551 cm^–1^ in the CS film shifted to 3,391, 1,645, and 1,570 cm^–1^ in the CS/WPI film, respectively, and similar observation has been reported by Tavares et al. (11). These results indicated that new covalent bonds and/or hydrogen bonds might be formed between WPI and CS molecules during the film formation process, thus leading to changes in the overall structure of the composite film, which was confirmed by improvements in mechanical properties (Table 1) and differences in the microstructure of the film (Figure 3).

With the addition of TiO_2_, the absorption bands at 3,391, 1,645, 1,570, and 1,058 cm^–1^ in CS/WPI film shifted to 3,393, 1,651, 1,575, and 1,055 cm^–1^ in CS/WPI/TiO_2_ film, respectively. This phenomenon indicated that the hydrogen bonds were formed between TiO_2_ nanoparticles and the polymer substrate (47, 48). The same results were observed by Alizadeh-Sani et al. (47) and Wang et al. (6) from chitosan/sodium alginate composite film incorporated with carboxymethyl chitosan-ZnO nanoparticles and cellulose nanofibre/WPI composite film incorporated with TiO_2_ nanoparticles. Moreover, the absorption band at 680 cm^–1^ was attributed to bending vibrations of Ti-O-Ti (18).

The peaks around 1,650 and 1,050 cm^–1^ were attributed to C = O and C-O stretching vibration of aldehyde and ester groups in EOs, respectively (49). Due to the hydrophobicity of WPEO, the intensity of the peak near 2,900 cm^–1^ increased in CS/WPI/WPEO film and CS/WPI/WPEO+TiO_2_ film, which was in consistent with the results of Hadidi et al. (25). The peaks at 3,392 cm^–1^ in CS/WPI/WPEO film and 3,394 cm^–1^ in CS/WPI/WPEO+TiO_2_ film became more flattened, which might be due to hydrogen bonding between the -OH group in EO and the -NH and -OH groups in CS (13).

#### Scanning electron microscopy

Surface and cross section of films were shown in Figure 3. The CS film showed smooth surface with compact structure, and smooth cross section without porous structure, indicating that CS had good film-forming property, and CS and glycerol were homogenously mixed. Similarly, the result was also reported by Wang et al. (12). The surface of CS/WPI film was dense and homogeneous, but slightly coarse, and its cross-section had compact structure without phase segregation and cracks. This might be attributed to the fact that the interaction between WPI and CS leads to the rearrangement of certain amino acids in the protein and the covalent cross-linking between molecules (5, 11).

Although several white TiO_2_ particles were observed on the surface of CS/WPI/TiO_2_ film, the overall structure of this film was dense without large aggregates. Our result was in accordance with those of Gohargani et al. (14), who evaluated the effect of TiO_2_ nanoparticles on the microstructure of chitosan/whey protein film. Its cross-sectional network structure exhibited good compactness without any pore, indicating TiO_2_ nanoparticles were well dispersed in the CS/WPI/TiO_2_ film, thus increasing the compactness of the film (37). The tight inner microstructure of CS/WPI/TiO_2_ film enhanced water vapor barrier property and mechanical properties as mentioned above.

The CS/WPI/WPEO film displayed the roughest surface, and its cross section had many visible pores, which might be due to the wrapped of EOs in a continuous network structure composed of polysaccharides and proteins, thus resulting in an uneven structure (22, 29). The hydrophobicity of the EO caused more holes in the composite film, thus destroying the structural integrity of the film. Consistently, Li et al. (37) have reported that adding turmeric EO to CS films increases the unevenness and roughness of CS films.

Our data showed that simultaneous incorporation of TiO_2_ and WPEO reduced the roughness and porosity of the film (Figure 3). The reason might be that the addition of TiO_2_ enhanced the interaction between polymers thus compensating for the unevenness of the film structure induced by the hydrophobicity of EOs. Zhang et al. (19) report that TiO_2_ reinforced the polymer matrix through electrostatic interactions, hydrogen bonding, or O–Ti–O bonding.

#### Water contact angle

**FIGURE 4:**
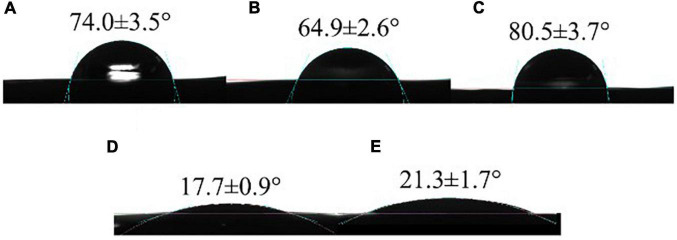
Water contact angles of CS **(A)**, CS/WPI **(B)**, CS/WPI/WPEO **(C)**, CS/WPI/TiO_2_ **(D)**, and CS/WPI/WPEO/TiO_2_ **(E)** films.

The WCA can be used to evaluate the hydrophobicity and surface wettability of packaging films (17). The WCA of different films was shown in Figure 4.

The WCA value of CS film was 70.4°, which was similar to Kurek et al. (50). The WCA was decreased when WPI was integrated into the film. The reason might be that the cross-linking reaction between CS and WPI exposed the carboxyl and hydroxyl groups of WPI, thus increasing the hydrophilicity of the composite film surface (11). CS/WPI/WPEO composite film exhibited the largest contact angle 80.5°, and the hydrophobicity of this film was increased due to the predominant non-polar substances in the EO (35). Similarly, Silveira et al. (51) reported that the WCA of cassava starch/cellulose nanofibre film incorporating tea tree EO increased from 54.2° to 84.5°. Xiong et al. (52) demonstrated that the increase of contact angle may be attributed to heterogeneous wetting resulting from the rough film surface. The surface of CS/WPI/WPEO film was rougher than the other films (Figure 4), potentially leading to the high contact angle. CS/WPI/TiO_2_ film had the minimum contact angle 17.7°, indicating that the significant enhancement of the hydrophilicity of the film due to the incorporation of highly hydrophilic TiO_2_ nanoparticles.

The contact angle of CS/WPI/WPEO+TiO_2_ film was approximately the similar to that of CS/WPI/TiO_2_ film, which significantly lower than that of CS/WPI/WPEO film, indicating that the addition of TiO_2_ considerably increased the hydrophilicity of the composite film. Although the enhanced hydrophilicity will reduce water resistance of the film, the enhanced hydrophilicity also facilitated the contact between the bacterial cells and the film, which contributed to increasing antimicrobial effects (17).

#### Antioxidant property

The antioxidant capacity is a vital index to evaluate the performance of food packaging films. As shown in Table 3, the IC_50_ of BHT in DPPH and ABTS radical scavenging assay were 0.09 and 0.11 mg/ml, respectively, which were consistent with those reported by Li et al. (31). The IC_50_ for WPEO was 0.159 mg/ml (DPPH) and 0.171 mg/ml (ABTS), respectively, which were slightly lower than that for BHT, indicating that WPEO had significant antioxidant properties. The DPPH and ABTS radical scavenging rates of CS film were 56.79 and 50.36%, respectively. The radical scavenging capacity of CS film might be attributed to the fact that the residual free amino groups of CS can react with free radicals to form stable macromolecular radicals and amino groups (53). However, CS film had lower antioxidant activity than other films.

**TABLE 3:** DPPH and ABTS free radical scavenging activity of WPEO and films (mg/ml).

Materials	Concentration (mg/ml)	DPPH		ABTS	
			
		Free radical scavenging activity (%)	IC_50_ (mg/ml)	Free radical scavenging activity (%)	IC_50_ (mg/ml)
CS	–	56.79 ± 0.36	–	50.35 ± 0.44	–
CS/WPI	–	62.66 ± 1.11	–	55.73 ± 0.57	–
CS/WPI/TiO_2_	–	74.75 ± 1.03	–	66.26 ± 1.71	–
CS/WPI/WPEO	–	85.56 ± 2.09	–	84.88 ± 1.80	–
CS/WPI/WPEO+ TiO_2_	–	90.84 ± 2.45	–	90.74 ± 3.98	–
WPEO	0.1	47.28 ± 3.05	0.159	45.04 ± 3.03	0.171
	0.2	55.6 ± 2.10		54.43 ± 1.32	
	0.4	69.33 ± 3.48		66.50 ± 2.76	
	0.8	86.17 ± 3.07		85.64 ± 2.88	
	1.0	91.35 ± 5.21		90.90 ± 4.49	
BHT		0.09		0.11	

The antioxidant activity of the films added with WPEO was significantly increased (*P* < 0.05), and the DPPH and ABTS radical scavenging activity of CS/WPI/WPEO film reached 86.60 and 84.89%, respectively. The increased antioxidant activity was attributed to the phenolic and terpenic compounds in EO, and these compounds could exert their antioxidant activity by multiple possible mechanisms, such as free-radical scavenging activity and hydrogen donors (25). Chen et al. (24) have also reported that the free radical scavenging activity of clove EO is associated with eugenol and β-caryophyllene. The main components of WPEO have been reported to be caryophyllene, 3-carene and D-limonene, and caryophyllene has been demonstrated to be the main reason for antioxidant activities of EOs (30, 31, 54). In our experiments, GC-MS analysis of WPEO also showed the highest concentration of caryophyllene at 0.12 mg/ml, followed by limonene and 3-carene at 0.076 and 0.073 mg/ml, respectively (Supplementary Table 1).

Our data showed the antioxidant activity of CS/WPI/TiO_2_ film was significantly increased (*P* < 0.05) to 74.76% for DPPH and 66.26% for ABTS. TiO_2_ can act as a reactive oxygen species (ROS) scavenger, and the free radical scavenging capability of TiO_2_ is due to the antioxidant properties of the nanoparticles (55). The antioxidant activity of CS/WPI/WPEO+TiO_2_ film was the highest, exceeding 90%, which indicated that TiO_2_ and WPEO had a synergistic effect on free radical scavenging.

#### Antibacterial property

**FIGURE 5:**
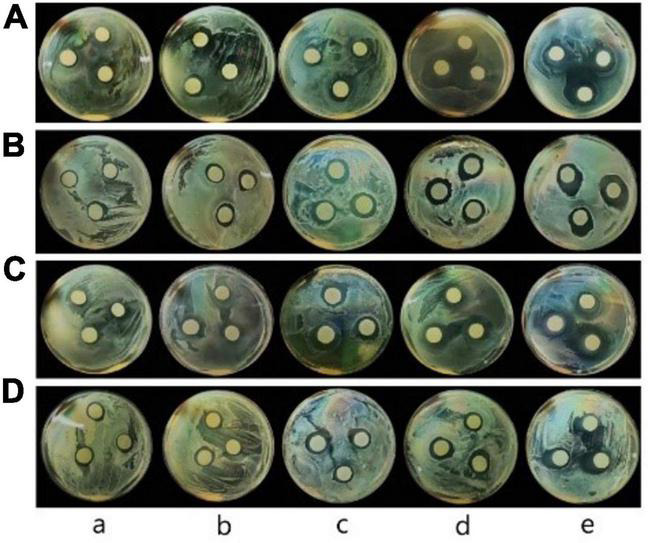
Photos of the inhibitory zone of CS (a), CS/WPI (b), CS/WPI/TiO_2_ (c), CS/WPI/WPEO (d), and CS/WPI/WPEO/TiO_2_ (e) films (**A:** *E. coli*; **B:** *L. monocytogenes*; **C:** *S. aureus*; **D:** *Salmonella*).

Inhibition zone diameters of films were shown in Figure 5 and Table 4. The inhibitory diameters of pure CS film against *E. coli*, *L. monocytogenes*, *S. aureus*, and *Salmonella* were 14.17, 15.30, 15.33, and 15.27 mm, respectively. No significant difference in bacterial inhibition was observed (*P* > 0.05) between CS/WPI film and CS film. The antibacterial activity of CS was attributed to the interactions between positive charges on chitosan and negative charges on microbial cell surface, thus resulting in the leakage of cytoplasmic content and death of the bacterial cells (6, 17).

**TABLE 4:** The inhibitory zone diameter of the composite films.

Films	Inhibitory zone diameter (mm)			
	
	*E. coli*	*L. monocytogenes*	*S. aureus*	*Salmonella*
CS	14.17 ± 0.20^d^	15.27 ± 0.34^c^	15.33 ± 0.34^b^	15.30 ± 0.29^c^
CS/WPI	14.67 ± 0.18^cd^	15.80 ± 0.37^c^	15.80 ± 0.24^b^	15.40 ± 0.16^c^
CS/WPI/TiO_2_	16.93 ± 0.42^bc^	17.20 ± 0.24^bc^	17.87 ± 0.17^b^	16.53 ± 0.25^bc^
CS/WPI/WPEO	17.80 ± 0.42^b^	19.10 ± 0.42^b^	22.00 ± 0.20^a^	18.10 ± 0.37^ab^
CS/WPI/WPEO/TiO_2_	20.83 ± 0.17^a^	21.50 ± 0.41^a^	25.00 ± 0.37^a^	19.73 ± 0.16^a^

Data are expressed as mean ± SD. Different letters (a–d) in each column indicate significant differences among films (*P* < 0.05).

Chitosan/WPI/TiO_2_ film showed stronger bacterial inhibition effect than CS and CS/WPI film. The possible reason lay in that TiO_2_ destroyed the covalent bonds of the peptidoglycan layer of the bacterial cell wall by generating ROS (16). This is also supported by another previous report that the surface positive charges of metal nanoparticles are prone to bind with the surface negative charge of bacteria, thus enhancing bactericidal effect (34).

The antibacterial activity of CS/WPI/WPEO film was significantly higher than that of CS and CS/WPI film (*P* < 0.05), and the inhibition diameter of CS/WPI/WPEO film against *E. coli*, *L. monocytogenes*, *S. aureus*, and *Salmonella* was 17.80, 19.10, 17.87, and 18.10 mm, respectively. The significant antibacterial property of WPEO was attribute to its high concentration of sesquiterpenes and monoterpenes such as caryophyllene, limonene and torreyol (31). Studies on the antibacterial effect of monoterpenes have demonstrated that they diffuse into the cell and damage the cell membrane (56).

The CS/WPI/WPEO+TiO_2_ film reached the highest antibacterial activity, in comparison with other films. Our data showed that the synergistic effect of WPEO and TiO_2_ resulted in stronger antibacterial properties of the composite film, and that Gram-positive bacteria, represented by *S. aureus* and *L. monocytogenes*, were more sensitive to the composite film added with WPEO than Gram-negative bacteria (*E. coli* and *Salmonella*). This might be due to the fact that the outer layer membrane of the Gram-negative bacteria contained large amount of lipopolysaccharide, which was relatively impermeable to lipophilic compounds (37, 57). Our results were consistent with several previous reports that the antibacterial activity of EOs against Gram-positive bacteria was significantly higher than that of Gram-negative bacteria (34, 39).

#### Release rate of white pepper essential oil

**FIGURE 6:**
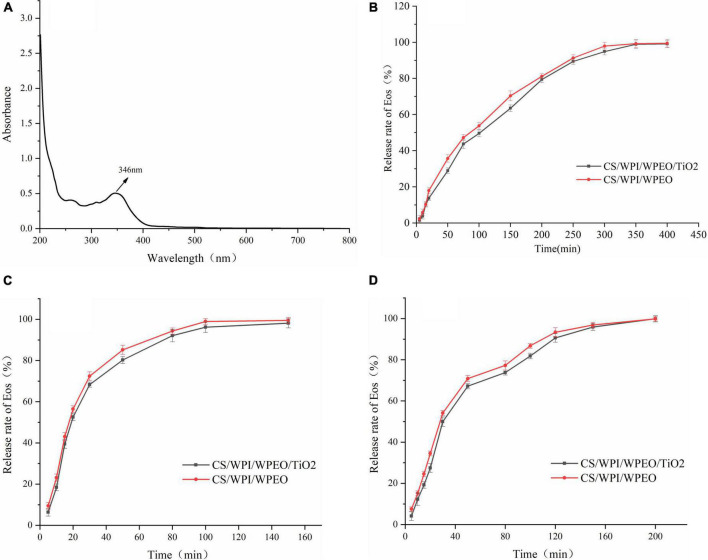
Full band scanning image of WPEO **(A)**. Release of WPEO from CS/WPI/WPEO and CS/WPI/WPEO/TiO_2_ films in 95% ethanol **(B)**, 50% ethanol **(C)**, and distilled water **(D)**.

The full wavelength scan of WPEO was shown in Figure 6A. The results showed that WPEO exhibited absorption peak at 346 nm. The absorbance of WPEO at 346 nm was measured in subsequent experiments.

Essential oils are volatile and can be lost during film formation and subsequent storage. As shown in Figures 6B–D, among the three food simulants, the release of WPEO from film in 95% ethanol (Figure 6B) was slow, and it took 400 min to reach the maximum release rate. It took 150 min and 200 min to reach the maximum release rate in 50% ethanol and distilled water, respectively with the maximum release rate in 50% ethanol. Our results were in line with the report by Lian et al. (36) that the release rate of thyme EO from CS film was slowest in 95% ethanol and fastest in 50% ethanol. This phenomenon might be due to the water molecule-induced film swelling and the solubility of EO in ethanol (37). It has been reported that in 50% ethanol, the release rate of EOs is accelerated because the film swelling facilitates opening the structure of the film network, which contributes to the diffusion of EOs (23).

In the early stage of release, water molecules enter into the films and lead to the swelling of films. Therefore, the structure of films becomes loose and the interactions between the compounds in the films are reduced, thus leading to the rapid release of the EO (27). Our data showed that WPEO was rapidly substantially transferred from the composite films to oil-in-water emulsions and alcoholic foods, but WPEO was released slowly into fatty foods, and that it could exert active long-time effect, which was consistent with the report by Liang et al. (58).

## Conclusion

According to this study, TiO_2_ nanoparticles and WPEO can be simultaneously incorporated into CS/WPI polymer matrix to synthesize bio-nanocomposite films. The results of FTIR and SEM showed that TiO_2_ was evenly incorporated in the films, which improved the mechanical properties and water vapor barrier properties. The incorporation of WPEO significantly enhanced the antioxidant and antibacterial properties of composite films. WPEO exhibited the slower release rate, thus it had a better effect in fatty food simulant than in the other two food simulants. The incorporation of TiO_2_ and WPEO had a synergistic effect, and they jointly significantly improved the water vapor barrier, UV-vis barrier properties, mechanical strength, antioxidant activity, and antibacterial properties, especially Gram-positive bacterium inhibition. This study showed that the composite film simultaneous adding TiO_2_ and WPEO is a potential packaging material.

## Data availability statement

The raw data supporting the conclusions of this article will be made available by the authors, without undue reservation.

## Author contributions

YiW: methodology, investigation, data curation, and writing—original draft. JW and JL: methodology and writing—review and editing. JW: software and investigation. XZ: visualization and resources. YZ and YoW: conceptualization, funding acquisition, supervision, and writing—review and editing. All authors have read and agreed to the published version of the manuscript.
