# Supplementary material for: Preparation and characterization of chitosan/whey isolate protein active film containing TiO2 and white pepper essential oil
Source: Front Nutr. 2022 Nov 21;9:1047988. doi: 10.3389/fnut.2022.1047988 (PMC9720319; doi:10.3389/fnut.2022.1047988)
Supplement: Supplementary file 1 [file Table_1.DOCX]

**Supplementary Material**

**Table S1.** Composition and content of volatile constituents in WPEO

| No. | CAS | Compounds | Retention index | mass concentration（mg/ml） |
| --- | --- | --- | --- | --- |
| 1 | [7785-26-4](https://www.chemsrc.com/baike/1118604.html) | α-Pinene | 948 | 0.0531 |
| 2 | [13466-78-9](https://www.chemsrc.com/baike/259575.html) | 3-Carene | 1035 | 0.0726 |
| 3 | [138-86-3](https://www.chemsrc.com/baike/509669.html) | Limonene | 1047 | 0.076 |
| 4 | [10579-93-8](https://www.chemsrc.com/baike/1621254.html) | Bicyclo[7.2.0]undec-4-ene, 4,11,11-trimethyl-8-methylene- | 1494 | 0.0679 |
| 5 | [95-48-7](https://www.chemsrc.com/baike/1036568.html) | o-Methylphenol | 1014 | 0.0558 |
| 6 | [99-86-5](https://www.chemsrc.com/baike/167571.html) | α-Terpinene | 998 | 0.0628 |
| 7 | [17627-40-6](https://www.chemsrc.com/baike/1279952.html) | Caryophyllene | 1494 | 0.1237 |
| 8 | [6753-98-6](http://www.ichemistry.cn/chemistry/6753-98-6.htm) | Humulene | 1579 | 0.0704 |
| 9 | [29350-73-0](http://www.ichemistry.cn/chemistry/29350-73-0.htm) | Cadinene | 1541 | 0.0718 |
| 10 | [17627-43-9](http://www.ichemistry.cn/chemistry/17627-43-9.htm) | Caryophyllene oxide | 1507 | 0.0687 |
| 11 | [77171-55-2](https://www.chemsrc.com/baike/808665.html) | (-)-Spathulenol | 1536 | 0.069 |
| 12 | [19670-49-6](http://www.ichemistry.cn/chemistry/19670-49-6.htm) | Octanoic acid, 2,3-dihydroxypropyl ester | 1687 | 0.0508 |
| 13 | [88395-46-4](https://www.chemsrc.com/baike/1656760.html) | Isospathulenol | 1536 | 0.0528 |
| 14 | 3376-48-5 | Decanoic acid, 2,3-dihydroxypropyl ester | 1886 | 0.0508 |
| 15 | 18836-52-7 | 2,4-Decadienamide, N-isobutyl-, (E,E)- | 1765 | 0.0575 |
| 16 | 28474-90-0 | l-(+)-Ascorbic acid 2,6-dihexadecanoate | 4765 | 0.0603 |
| 17 | 1678-45-1 | Dodecanoic acid, 2-hydroxy-1-(hydroxymethyl)ethyl ester | 2101 | 0.0698 |
| 18 | 5422-81-1 | Piperidine, 1-(1-oxo-3-phenyl-2-propenyl)- | 1840 | 0.0531 |
| 19 | 78910-33-5 | 2,4-Decadien-1-one, 1-(1-pyrrolidinyl)-, (2E,4E)- | 1759 | 0.0545 |
| 20 | 88855-41-8 | 9-Octadecenoic acid | 2175 | 0.0564 |
| 21 | 112248-30-3 | Eicosen-1-ol, cis-9- | 2260 | 0.0597 |
| 22 | 3443-83-2 | Tetradecanoic acid, 2-hydroxy-1-(hydroxymethyl)ethyl ester | 2300 | 0.0639 |
| 23 | 60514-48-9 | 1,2-Dioctanoin | 2423 | 0.0449 |
| 24 | 119-47-1 | Phenol, 2,2'-methylenebis[6-(1,1-dimethylethyl)-4-methyl- | 2788 | 0.0589 |
| 25 | 23470-00-0 | Hexadecanoic acid, 2-hydroxy-1-(hydroxymethyl)ethyl ester | 2498 | 0.0692 |
| 26 | 93980-84-8 | Decanoic acid, 2-hydroxy-3-[(1-oxooctyl) oxy] propyl ester | 2606 | 0.0575 |
| 27 | 693-80-1 | 9-Octadecen-1-ol, acetate | 2185 | 0.05 |
| 28 | 93980-84-8 | Decanoic acid, 2-hydroxy-1,3-propanediyl ester | 2804 | 0.0522 |

## Analysis of WPEO composition

The WPEO composition was analysed by the method of Amalraj et al. (1) with minor modifications.100 μL of essential oil was mixed with 25 µL of cyclohexanone at 0.05 mg/mL as internal standard and fixed with hexane to 5 mL. The analysis was performed by GC-MS (Trace ISQ, Thermo Fisher Scientific Co. The temperature was held at 90°C for 2 minutes, then set the first column temperature ramp to 150°C at a rate of 8°C/min and maintained for 10 minutes, and set the second column temperature ramp to 230°C at a rate of 5°C/min and increased the final temperature to 280°C at a rate of 20°C/min and kept for 15 minutes. Helium was used as the carrier gas at a 1 mL/min flow rate and a 1 µL injection volume without splitting. WPEO components were identified using the National Institute of Standards and Technology - Mass Spectrometry Search Library version 2.0 (NIST 14).

## Reference

(1) Amalraj, A., Haponiuk, J.T., Thomas, S., Gopi, S. (2020). Preparation, characterization and antimicrobial activity of polyvinyl alcohol/gum arabic/chitosan composite films incorporated with black pepper essential oil and ginger essential oil. Int J Biol Macromol 151, 366-375. https://doi.org/ 10.1016/j.ijbiomac.2020.02.176.
